# Serum Tumor Markers in Pancreatic Cancer—Recent Discoveries

**DOI:** 10.3390/cancers2021107

**Published:** 2010-06-02

**Authors:** Felix Rückert, Christian Pilarsky, Robert Grützmann

**Affiliations:** Department of Visceral-, Thoracic- and Vascular Surgery, University Hospital Carl Gustav Carus, Technical University Dresden, Germany; E-Mails: christian.pilarsky@uniklinikum-dresden.de (C.P.); robert.grützmann@uniklinikum-dresden.de (R.G.)

**Keywords:** pancreatic cancer, serum tumor markers

## Abstract

The low prevalence of pancreatic cancer remains an obstacle to the development of effective screening tools in an asymptomatic population. However, development of effective serologic markers still offers the potential for improvement of diagnostic capabilities, especially for subpopulations of patients with high risk for pancreatic cancer. The accurate identification of patients with pancreatic cancer and the exclusion of disease in those with benign disorders remain important goals. While clinical experience largely dismissed many candidate markers as useful markers of pancreatic cancer, CA19-9 continues to show promise. The present review highlights the development and the properties of different tumor markers in pancreatic cancer and their impact on the diagnostic and treatment of this aggressive disease.

## 1. Introduction

Pancreatic cancer (PDAC) is an exceptionally devastating and in most cases incurable disease with surgery as the only treatment with curative intent. Five-year survival rates are approximately 20% for patients undergoing potentially curative resection. However, postoperative disease recurrence occurs commonly. Unfortunately, only 10–20% of patients show a resectable tumor at the time of diagnosis [1]. The poor overall results of current conservative treatment for pancreatic cancer in contrast with the five-year survival following resection of tumors suggested that better results might be achieved if patients could be identified earlier by screening test, e.g., tumor markers. 

However, early enthusiasm about screening possibilities of serum tumor markers was not always justified. Today, serum tumor markers should only be used for the monitoring of malignant diseases. Only in very few exceptions the use of tumor markers for screening is reasonable, e.g., PSA for prostate cancer. The reason for this is based on the relative low incidence of neoplasms in a normal population. This can be exemplified for pancreatic cancer: PDAC has a prevalence of 6/100,000 in the normal population [1]. If such a population of 100,000 were screened by a tumor marker with a sensitivity of 100% and a specificity of 99%, there would be 1010 positive tests of which only six would be true positives and the remaining 1004 would be false positives. All these patients would then have to be subjected to further tests including imaging and even invasive diagnostic. 

Of course in certain subgroups, like patients with hereditary pancreatitis, the incidence of PDAC is higher. Therefore, screening with tumor markers in these groups can be reasonable [2,3]. For the same reason, tumor markers can provide an important improvement in the diagnostic by discriminating pancreatic cancer among symptomatic patients with various gastrointestinal disorders- especially chronic pancreatitis- in whom there is a high suspicion of malignancy. Moreover, when pancreatic cancer is proven histologically, tumor markers can be valuable tools in the follow-up of the disease as already mentioned above. This paper gives an overview of established and experimental serologic markers in the diagnosis of human pancreatic cancer. We also try to weight the acceptability of each marker for clinical and experimental application by review of literature. 

## 2. Serum Tumor Markers for Pancreatic Cancer

A wide variety of serologic markers have been associated with pancreatic cancer. These may be broadly divided into four groups: tumor-associated antigens, enzymes, oncofetal antigens and others, including the ectopic production of hormones and other peptides [2,4]. Unfortunately, many putative markers have failed to bear out their initial promise. Today, the most widely used markers are tumor associated antigens. 

### 2.1. Tumor Associated Antigens

The hybridoma technology, with its availability of monoclonal probes, has been an important step for the establishment of tumor markers. Many tumor-associated antigens have been defined by this immunologic technique [4]. Tumor-associated antigens (TAA) used as tumor biomarkers in pancreatic cancer can be molecularly defined as carbohydrate antigens, glycoproteins, mucins and cytokeratins. The most widely studied TAA in pancreatic cancer is the carbohydrate antigen CA19-9. Carbohydrate antigens, but also other TAA, are elevated in obstructive jaundice. The mechanism by which cholestasis increases levels of those markers is still obscure, but it is proposed that as a consequence of secondary bile salt damage and inflammation, bilio-pancreatic ducts might be damaged and tumor antigens be released from the epithelium [3]. 

#### 2.1.1. Carbohydrate Antigens

Carbohydrate antigens such as CA19-9, CA50, CA125, and CA242 contain oligosaccharide structures present on heavily glycosylated high molecular weight mucins [5]. 

##### 2.1.1.1. CA19-9

Colon specific antigen, a predominantly carbohydrate antigen, was the initial name given to CA19-9. The CA19-9 antigen is defined by an IgG1 mouse monoclonal antibody raised against the human colonic carcinoma cell line SW 1116 by Koprowski and colleagues in 1979 [6]. CA19-9 has a half-life of four to eight days, its epitope has been shown to be the sialylated Lewis antigen [3]. CA19-9 was initially detected in colorectal cancer tissue but has been found more widely distributed in normal pancreas, stomach, and biliary epithelium. Normal adult pancreas has been observed to express CA19-9 in about 80% of cases, usually in the apical border of ductal cells and often more strongly in large ducts, whereas acinar structures and Langerhans islets are negative [6]. Studies showed that CA19-9, as with many other tumor markers, is predominantly carried in serum by a mucus glycoprotein [7]. Although the CA19-9 antibody was generated against a colorectal cancer cell line, it is found more frequently in the sera of patients with pancreatic carcinoma than in colorectal or stomach carcinoma [8]. This probably reflects the propensity of pancreatic cancer to cause back-secretion of mucin into the blood rather than differential expression of the epitope [3]. Although CA19-9 is not accurate enough to be used in screening asymptomatic subjects for pancreatic cancer, it is currently the single most useful blood test in differentiating pancreatic cancer from chronic or recurring pancreatitis with a sensitivity ranging from 70–90% and a specificity from 68–91% (Table 1) [9,10,11,12]. It is also one of the most significant prognostic factors for both patients with resectable and those with unresectable disease [13,14,15]. Measurement of CA19-9 as a prognostic factor provides valuable information to assist in the therapeutic decision making especially for surgeons, because early recurrence can be expected in patients with high preoperative levels of the markers. An elevated tumor marker value even after resection indicates the high possibility of remnant disease [16]. Although the measurement of the tumor size by imaging is standard for evaluation in response to non-surgical treatments such as chemotherapy and radiotherapy, change in CA19-9 assists the evaluation practically because of the difficulty in accurate measurement of pancreatic mass with obscure margin in most patients, and because of high incidence of the clinically occult progression associated with this disease [17,18]. 

It has been shown that elevated serum concentrations of CA19-9 decrease after curative surgery and conversely that recurrent disease is often associated with an increase in the circulating level of this serum marker, which is a prerequisite for the use in follow-up of PDAC [9]. 

The diagnostic value of CA19-9 is limited in obstructive jaundice, where CA19-9 binding has been reported in up to 28% of patients [6]. Furthermore, CA19-9 values are of limited use in distinguishing mucinous neoplastic lesions like mucinous cystic tumors and intraductal papillary mucinous tumors from mucinous lesion with benign features [12]. 

In conclusion, CA19-9 is not sufficient for screening patients with pancreatic cancer. However, it has advantage in differential diagnosis between PDAC and chronic pancreatitis, assisting the assessment of treatment response, follow-up of pancreatic cancer and prognosis. 

**Table 1:** Summary of results for diagnostic pancreatic tumor markers.

Type of marker	Author	Sensitivity	Specificity	Patients tested (n)
CA19-9	Steinberg [13]	81	90	meta
	Goonetilleke [14]	79	82	meta
CA50	Kobayashi [15]	84	85	200
	Jiang [19]	78	70	129
CA242	Banfi [20]	57	93	41
	Jiang [19]	82	78	129
	Ni [21]	60	76	68
CA195	Banfi [20]	76	85	41
	Andicoechea [22]	82	73	67
CA125	Haglund [23]	45	76	95
	Duraker [24]	57	78	123
PAM4	Gold [25]	77	95	53
TAG-72	Pasquali [26]	45	95	58
CEA	Ni [21]	45	75	68
	Haglund [23]	54	76	95
	Duraker [24]	39	91	123
	Zhao [27]	25	86	143
POA	Nishida [28]	81	96	21
	Zhao [27]	68	88	143
TPA	Panucci [29]	96	67	28
	Benini [30]	48	80	25
	Pasanen [31]	52	85	25
TPS	Banfi [20]	98	22	41
	Pasanen [32]	50	70	26
Du-PAN 2	Satake [33]	48	85	239
	Sawabu [34]	72	94	32
	Kawa [35]	64	-	200
SPan-1	Kiriyama [36]	81	76	64
	Chung [37]	92	83	67
	Kobayashi [15]	82	85	200
CAM17.1	Parker [38]	78	76	79
	Gansauge [39]	67	100	91
TATI	Taccone [40]	92	67	36
	Pasanen [41]	41	63	17
	Aroasio [42]	63	-	52
Elastase-1	Zhao [27]	62	67	143
GT II	Uemura [43]	77	85	13
Tu M2-PK	Ventrucci [44]	85	41	60
	Cerwenka [45]	79	90	38
	Oremek [46]	71	95	64
Mic-1	Koopmann [47]	90	94	50
	Koopmann [48]	71	78	80

Previous studies of serum tumor markers in pancreatic cancer (meta = meta-analysis, GT II = Galactosyltransferase isoenzyme II, Tu M2-PK = Tumor M2-Pyruvate Kinase).

##### 2.1.1.2. CA50

**Figure 1:**
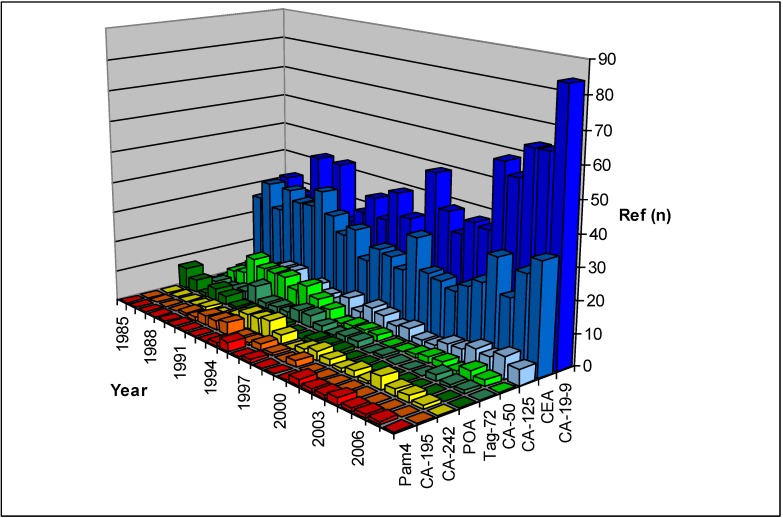
Results of the Pubmed search for references on carbohydrate antigens and glycoproteins in pancreatic cancer serum markers.

The tumor marker CA50 is defined by the murine IgM type monoclonal antibody C 50. This antibody was developed against the colorectal cancer cell line COLO205 by Lindholm and colleagues in 1983 [2]. Like CA19-9, its epitope has been identified on both a monosialoganglioside and a sialylated glycoprotein in colorectal and pancreatic tumor tissue [3]. CA50 binding in serum has a sensitivity and specificity comparable to CA19-9 (Table 1). The interference of cholestasis with the CA50 assays is only commented on in a few reports [49]. However, when interpreting the serum CA50 values, the effects of jaundice and cholestasis must be considered, as they may reduce the diagnostic specificity for cancer. Our review of literature shows that interest in CA50 was highest in the late eighties, while today it is only sporadically cited (Figure 1). 

##### 2.1.1.3. CA242

CA242 antigen is defined by the monoclonal antibody C-242, which was obtained by immunizing mice with the human colorectal carcinoma cell line COLO205. The chemical structure of the antigenic determinant is not exactly described, but it seems to be a sialylated carbohydrate structure [3]. CA242 is related, but not identical, to the epitope of CA19-9 and CA50 [50]. Increased serum levels of CA242 occur most frequently in patients with pancreatic and colorectal carcinomas [51]. The reported overall sensitivity and specificity of the assay for pancreatic cancer is inferior to CA19-9 or CA50 (sensitivity 57–82%; specificity 76–93%) (Table 1). The interference of cholestasis and jaundice with the CA242 assays is commented on in some studies [49]. Because of these circumstances, CA242 never experienced a widespread application in clinical and experimental use (Figure 1).

##### 2.1.1.4. CA195

The tumor marker CA195 was obtained by immunizing mice with a membrane preparation from liver metastases of a human colonic cancer [52]. Epitope analysis has shown that the monoclonal antibody binds to sialylated Lewis antigen, as the monoclonal antibody C19–9, but also to the Lewis glycolipid antigen itself [53]. The reported overall sensitivity and specificity levels of CA195 for diagnosis of pancreatic cancer are inferior to CA19-9 (Table 1) [6]. As seen in Figure 1, CA195 recently has not been used in experimental studies in pancreatic cancer. 

##### 2.1.1.5. CA125

Cancer marker CA125 is a high-molecular weight glycoprotein defined by a monoclonal antibody raised against an ovarian cystadenocarcinoma cell line. It has been shown to be identical to the gene product of MUC16 of the MUC protein family [5]. CA125 is expressed in epithelial ovarian cancer tissue and also in human pancreatic cancer [54]. Serum analysis of CA125 in diagnosis of pancreatic cancer shows poor sensitivity and specificity (Table 1). The clinical usefulness of CA125 in diagnosis of pancreatic cancer is further limited, because 64% of patients with liver cirrhosis, 23% of patients with hepatitis, 25%–38% of patients with pancreatitis, and 35% of patients with jaundice also have increased levels of CA125 [8,55] (Figure 1).

##### 2.1.1.6. Other Carbohydrate Antigens

CA494 was initially isolated from mice immunized with a human colon cancer cell line. The exact structure of the CA494 antigen is unknown [8,56]. Although initial studies were promising with 90% sensitivity at a 94% specificity [57], we did not see clinical application of this marker. Further markers aiming at carbohydrate antigens are experimental markers PAM4 [25] and TAG-72 [58] (Figure 1). 

#### 2.1.2. Glycoproteins

Glycoproteins are proteins that contain oligosaccharide chains covalently attached to their polypeptide side-chains. The carbohydrate is attached to the protein in a cotranslational or posttranslational modification. Glycoproteins are often important integral membrane proteins, where they play a role in cell-cell interactions [55].

##### 2.1.2.1. CEA

Carcinoembryonic antigen (CEA) was first described in 1965 by Gold and Freedman after immunization of rabbits with an extract of human colon carcinoma. CEA is a glycoprotein with a molecular weight of 180 kDa, with branched oligosaccharide chains linked to a polypeptide chain. The major antigenic epitopes are localized on the protein part, and at least six different epitopes are identified [56]. CEA is normally present during fetal life in the liver, pancreas, and gastrointestinal tract and in adolescence in small amounts in the colon and endodermal tissues. Its name is a misnomer, as low concentrations of CEA are found in many normal adult tissues as well as fetal tissue [3]. CEA was for more than a decade the only serum tumor marker used clinically in the diagnosis of pancreatic cancer. During the past 25 years, CEA has been replaced by other markers, which have shown a higher sensitivity. There are many reports of its analysis in pancreatic cancer sera with a wide range of reported sensitivities (25–54%) and specificities (75–91%), with an unacceptably low sensitivity for clinical use [2,8] (Figure 1).

##### 2.1.2.2. POA

In the early 1960s, Hobbs and colleagues developed polyclonal antibodies against fetal pancreas for the use in the detection of serum oncofetal antigens in pancreatic cancer. Early results were very promising [3]. However, because the antigen is detected by polyclonal antibodies, which recognize a range of epitopes, the assay is very difficult to standardize. The great variation in sensitivity (68–81%) (Table 1) of the POA serum test is obviously a problem in the routine use of this serum tumor marker in pancreatic cancer. According to present studies, it is obvious that the POA marker test hardly has a place in the work-up aimed at early diagnosis of pancreatic cancer (Figure 1) [2,6]. POA is frequently pathological in hepato-biliary diseases, which further limits the value of this marker in the diagnosis of pancreatic cancer [59]. 

#### 2.1.3. Cytokeratins

Cytokeratins are proteins of keratin-containing intermediate filaments found in the intracytoplasmic cytoskeleton of epithelial tissue. The expression “cytokeratin” was first used in the late 1970s [60]. Upon release from proliferating or apoptotic cells, cytokeratins provide useful markers for epithelial malignancies, distinctly reflecting ongoing cell activity [61].

##### 2.1.3.1. TPA

**Figure 2:**
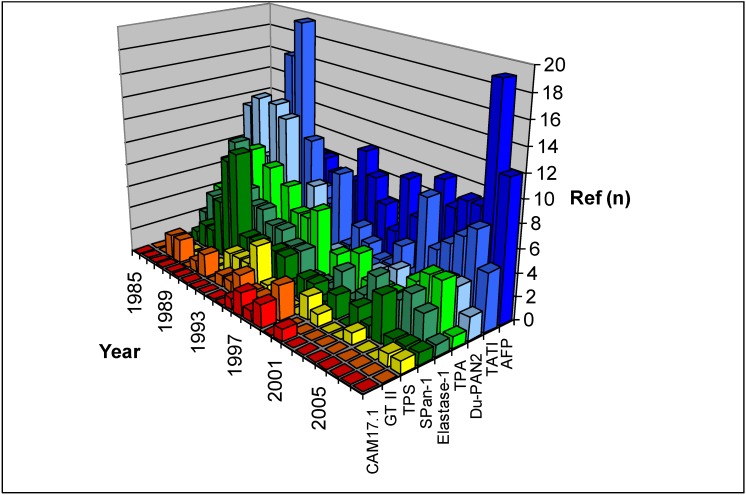
Results of the Pubmed search for references on cytokeratins, mucins and others in pancreatic cancer serum markers (GT II= Galactosyltransferase isoenzyme II).

Tissue polypeptide antigen (TPA) was first described in 1957 by Björklund [62]. TPA is a broad spectrum test that measures cytokeratins 8, 18, and 19 [61]. The release of this antigen is a function of cell division; therefore it differs from many other tumor marker tests and possibly indicates the tumor proliferative rate rather than the tumor burden. Its diagnostic value has been reported to be slightly inferior to that of CA50 and CA19-9, with a sensitivity of 48–96% (Table 1). TPA never saw a breakthrough in clinical application, although it was proposed that TPA might have a complementary role together with other serum tumor markers because of its different nature [6] (Figure 2). 

##### 2.1.3.2. TPS

The TPS assay is more specific and measures cytokeratin 18 [61]. It is produced during late S and G2 phases of the cell cycle and released immediately after mitosis, and increased serum levels may reflect malignant growth [6]. In the literature, few data are available on the utility of serum TPS assay in the diagnosis of pancreatic cancer, but the sensitivity (50–98%) and specificity (22–70%) of serum TPS in the diagnosis of pancreatic cancer seem inferior to CA19-9 (Table 1). 

#### 2.1.4. Mucins

Mucins are high molecular weight glycoproteins*.* Mucus glycoproteins are often present in the sera of patients with pancreatic cancer, and their detection and quantification can be used in serologic diagnosis [36]. Mucins are synthesized either as membrane bound or as secreted glycoproteins. The structure of epithelial mucins displays a protein backbone bearing numerous carbohydrate side chains [5]. 

##### 2.1.4.1 Du-PAN 2

Du-PAN 2 is a monoclonal antibody raised against the human pancreatic adenocarcinoma cell line HPAF. The antibody has been shown to recognize an antigen present on some cells of fetal pancreas and on ductal epithelial cells of normal adult pancreas, as well as on adenocarcinoma cells of pancreatic and non-pancreatic origin [63]. It is of particular interest that this antibody detects a different antigen from CA19-9 as it is able to detect a tumor marker in Lewis a-b-individuals [64]. The diagnostic sensitivity of the serum DU-PAN 2 marker is inferior to CA19-9 (48–72%). However, Du-PAN 2 still experiences application in experimental studies, especially in the Asiatic region (Table 1) (Figure 2).

##### 2.1.4.2 SPan-1

SPan-1 monoclonal antibody was produced by immunization with the mucin-producing human pancreatic cancer cell line SW1990 and reacts with a sialylated epitope carried by a mucin [37]. SPan-1 was initially considered as an additional useful and reliable serum marker for the detection of PDAC, with a sensitivity of 82–92%, but it does not significantly improve the diagnostic accuracy obtained with CA19-9 [65]. Furthermore, the marker is not specific for chronic liver diseases [36]. The usefulness of this marker might be underestimated, because only few studies considered this marker (Figure 2). 

##### 2.1.4.3. CAM17.1

CAM17.1 antibody was first described in the mid-90s as a monoclonal antibody that was generated after immunization with the colorectal cancer cell line Coll 2–23 [36]. CAM17.1 detects a mucus glycoprotein with high specificity for intestinal mucus. CAM17.1 is weakly expressed on normal ductal cells and chronic pancreatitis, whereas it is overexpressed in tissue specimens of pancreatic cancer [39]. Initial studies found a sensitivity of 78% and specificity of 76% for CAM17.1 as a serum marker in patients with pancreatic cancer [38]. However, recently there have been no studies regarding this marker in PDAC. 

### 2.2. Enzymatic Proteins

Enzymes represent a second group of potential tumor markers. This group includes enzymatic activities normally present in pancreatic tissue that may increase in the presence of cancer, e.g., ribonuclease, amylase, and alkaline phosphatase, as well as more tumor-specific isoenzymes such as Tumor-associated trypsin inhibitor and galactosyltransferase isoenzyme II. Most of the pancreatic enzymes like amylase, pancreatic isoamylase, lipase and trypsinogen showed no consistent alteration in serum concentrations in pancreatic cancer [66]. However, some enzymes were considered as serum markers in pancreatic cancer. 

#### 2.2.1. TATI

TATI is a 6000-Da peptide produced by several tumors and cell lines. It was shown to be identical to pancreatic secretory trypsin inhibitor [41]. Studies with regard to the utility of serum TATI as tumor marker in pancreatic cancer showed varied results (Table 1). As seen in Figure 2, interest in TATI was highest in the early 90s of the last century, with sensitivities ranging from 41–92%. Most of the recent publications concerning TATI are reviews. The decrease in interest in TATI might be due to its low specificity (58–67%), since increased values have been detected in many patients with benign pancreatic disease [42,67]. 

#### 2.2.2. Tumor M2-Pyruvate Kinase

An isoenzyme of pyruvate kinase (Tu M2-PK) is overexpressed by tumor cells and can be measured in blood by a specific immunoenzymatic assay [43]. The sensitivity (71–85%) and specificity (41–95%) of Tu M2-PK for pancreatic cancer is inferior to CA19-9. However, used together with CA19-9, the sensitivity increases considerably [44,45]. A benefit of Tu M2-PK might be that cholestasis appeared not to affect the values of Tu M2-PK [44] and that Tu M2-PK showed better correlation to metastasis than CA19-9 and CEA [45]. However, Tu M2-PK was also elevated in 64.3% of the patients with benign pancreatic pathologies, which might narrow the interest of clinicians in this marker [45]. 

#### 2.2.3. Elastase-1

Ventrucci *et al.* found significant elevation of serum concentrations of elastase-1 in pancreatic cancer [66]. However, further studies showed generally poor sensitivity and specificity [27,68]. Today, elastase-1 is only rarely used for experimental or clinical studies (Figure 2). 

#### 2.2.4. Galactosyltransferase Isoenzyme II

Glycosyltransferases are membrane-bound enzymes involved in glycoprotein and glycolipid synthesis. Along with other cell membrane components they are continually shed from the cell membrane. Tumor cells commonly shed glycosyltransferases [43]. In pancreatic cancer Galactosyltransferase isoenzyme II (GT II) shows low specificity (85%) and sensitivity (77%). Because of its frequent elevation in hepato-biliary diseases it seems to be of limited value in the diagnosis of pancreatic cancer [59] (Figure 2).

### 2.3. Oncofetal Antigens

Oncofetal antigens comprise tumor-associated antigens present in fetal tissue but not in normal adult tissue, such as alpha-fetoprotein, pancreatic oncofetal antigen and carcinoembryonic antigen. Those antigens were frequently developed by immunizing animals with tissue preparations. Because of that, most antibodies are polyclonal. 

#### 2.3.1. AFP

Alpha-fetoprotein was first recognized as serum marker for hepatocellular carcinoma by Abelev in 1963. In addition, elevated levels have also been noted in a small number of patients with extensive hepatic metastases. However, AFP appears to be of limited value in the diagnosis of pancreatic cancer [2]. McIntire found elevated AFP levels in 24% of patients with pancreatic cancer, and even lower sensitivity has been noted in other series [69,70]. However, AFP has seemingly a role in detection of rare tumors of the pancreas like acinar cell tumor [71] and pancreatoblastoma [72,73] (Figure 2). 

### 2.4. Other Tumor Markers

A wide variety of other markers have been studied, e.g., hormones, which did not show sufficient sensitivity or specificity for clinical use. However, there are also some promising new candidates as serum biomarkers for pancreatic carcinoma [2]. 

#### 2.4.1. EPM-1

Common epithelial cell surface marker (EPM-1) was defined by two monoclonal antibodies against the pancreatic tumor cell line Capan-1. The antigen is detectable in serum as a 400 kDa glycoprotein [3]. EPM-1 is an intriguing marker because, unlike all other markers, it is present in the serum of normal individuals but falls to low or undetectable levels in patients with gastrointestinal malignancy [74]. In pancreatic cancer, EPM-1 still has experimental status. 

#### 2.4.2. OPN

Initial studies showed a sensitivity and specificity of elevated osteopontin (OPN) for pancreatic cancer of 80% and 97%, respectively [75]. However, OPN did not provide additional diagnostic power together with CA19-9 in the differentiation of patients with resectable pancreatic cancer from controls [47]*.* Recently, there were no studies investigating the role of OPN in PDAC. 

#### 2.4.3. CEACAM1

CEACAM1 (CD66a; biliary glycoprotein) is a member of the carcinoembryonic antigen (CEA) family and of the immunoglobulin superfamily [69]. A previous report described an elevation of CEACAM1 in the sera of 91% (74/81) of pancreatic cancer patients, 24% (15/61) of normal patients, and 66% (35/53) of patients with chronic pancreatitis, with a sensitivity and specificity superior to CA19-9. To assess the value of this marker in the diagnostic of pancreatic cancer, further studies would be advantageous [70].

#### 2.4.4. MIC-1

Macrophage inhibitory cytokine 1 (MIC-1) encodes a protein that bears the structural characteristics of a transforming growth factor beta (TGF-beta) superfamily cytokine [76]. A previous study showed MIC-1 to be a significant independent predictor in the diagnosis of PDAC. However, although analysis showed that MIC-1 was significantly better than CA19-9 in differentiating patients with pancreatic cancer from healthy controls this marker could not distinguish pancreatic cancer from chronic pancreatitis [47]. 

## 3. Discussion

Although there were reports on numerous promising tumor markers in experimental settings, further studies for the clinical application of tumor markers for pancreatic cancer were often disappointing. There are several reasons for this. Firstly, in pilot studies, the patient cohort tends to have advanced disease, with the control group drawn from the young and healthy. Subsequent studies are often conducted on clinically more appropriate patient and control group. Secondly, the disease prevalence in the studies is commonly 50%. Hence, the positive predictive value is high [71]. 

Most tumor markers show a low sensitivity and specificity in the early stage of pancreatic carcinoma and are not appropriate for detecting early cancer [77]. Until today, there is no tumor marker apart from CA19-9, which experienced broad clinical application. Even CA19-9 shows an unsatisfactory sensitivity and specificity. 

Also, the concept of combining existent tumor makers to a panel of biomarkers was not trend-setting. Although this concept was supported by several studies, no such panel could be identified [78]. 

Because curative resection is only possible in an early stage of PDAC, the search and the establishment of new tumor markers is elementary. A prerequisite for the identification of tumor markers is the assumption that tumor-specific markers are specifically upregulated in PDAC or the tumor microenvironment [79] and may be found in the patients’ circulation. New high-throughput technologies like gene expression analysis [80,81] or protein profiling [82,83] greatly promoted this field of research. Recently, different groups tried to assemble and assess the great quantities of new potential tumor markers [84]. 

However, even if tumor-specific overexpressed candidates are identified, those might not be elevated in the patients’ circulation. Our own studies showed that even strongly upregulated, secreted proteins might not be detectable in the circulation [85]. This circumstance might be due to the specific pathophysiology and micro-architecture of the tumor. PDAC is not only poorly perfused and poorly vascularized, but also embedded in a prominent stromal matrix, which might detain candidate markers to trespass into the circulation [86]. An interesting new strategy to circumvent this problem is the evaluation of autoantibodies against aberrant or overexpressed autoantigens induced during the development of PDAC for the use as a tumor biomarker [87]. 

Hopefully, further studies will come up with a sensitive and specific marker. This would be a great benefit for patients by reducing time of diagnosis, by better estimating prognosis and earlier detection of recurrence.

## 4. Conclusions

Tumor markers seemed to be ideal for early diagnosis of cancer. However, the lack of sensitivity and specificity has been a major problem in the use of most serum tumor markers for diagnosis of pancreatic cancer. In the vast majority of research studies over the past two decades, CA19-9 alone has been applied as the ‘gold standard’ for monitoring and diagnosis of patients with pancreatic cancer. The recent advances of knowledge in the molecular biology of pancreatic cancer will hopefully result in serum markers with a diagnostic accuracy higher than CA19-9. 
